# Muscular Rigidity in Acute Peritonitis

**Published:** 1900-06-16

**Authors:** 


### MUSCULAR RIGIDITY IN ACUTE PERITONITIS.

Mr. Stanmore Bishop[1] has been digging deeply into the works of leading authorities in the hope of finding some definite guidance by which to ascertain whether in any given case peritonitis be present or absent. And he has been disappointed. Given a patient complaining of sudden and great pain in the abdomen, we should be able to say at once, and without hesitation, whether he or she is suffering from acute peritonitis or not, and he asks, Can we do so? He finds the teachings of the text-books on this important subject "vague." To show the sort of thing which pervades the text-books, Mr. Bishop quotes the following sentence: "In mechanical obstruction of the bowels the temperature is, as a rule, not above normal, unless complications have set in; while in peritonitis a rise of temperature is the rule, although in some of the gravest cases it is subnormal"; and he adds, "the fact may be true enough, but . . . I defy any man to make out from such a statement whether in any given case any reliance is to be placed on the temperature or not." Having asked a question, however, Mr. Stanmore Bishop does not hesitate to answer it, and he gives as the one invariable and absolute sign of acute peritonitis "rigidity of the abdominal muscles." "With acute peritonitis there is always rigidity of the abdominal muscles. Without peritonitis there is no rigidity." So far as it goes this is a useful "tip," but it must be taken with the limitations by which it is defined. The rigidity must be real. "The surgeon who goes to his patient's bedside, flings down the coverings, and places a cold, possibly wet, hand suddenly upon his patient's abdomen, and relies on this sign, will find all his patients suffering from this condition. But they are not. Such stiffening of the abdominal wall as he will meet with is not true rigidity, it is spasmodic contraction of the recti. The patient is not suffering from peritonitis; she is suffering from a totally different complaint, and its proper name is Dread of the Doctor. . . . The hand must not be suddenly applied, nor should it be used in the standing position. The surgeon must find time to sit down. His hand must be warm, warmer, if possible, than the patient's skin. . . . The entire hand must come in contact with the abdomen like a feather, so that it is almost impossible for the patient to say when actual contact is made. It should then lie without any weight, almost entirely supported by the surgeon's own muscles, for a few moments motionless, then gradually and gently move with an imperceptible sliding action over the surface. Thus, and thus only, will he be qualified to say whether or not there is actual muscular rigidity."

[1] Lancet, June 9.

